# Radioiodine-induced cGAS-STING signaling and antitumor immunity in thyroid cancer

**DOI:** 10.3389/fimmu.2026.1985864

**Published:** 2026-09-30

**Authors:** Feng Gao, Daoming Zhang, Yan Luo, Chunhua Li

**Affiliations:** 1 Department of General Surgery, Affiliated Hospital of Changchun University of Chinese Medicine, Changchun, China; 2 Patient Services Center, Affiliated Hospital of Changchun University of Chinese Medicine, Changchun, China; 3 Department of Endocrinology and Metabolism, Affiliated Hospital of Changchun University of Chinese Medicine, Changchun, China

**Keywords:** antitumor immunity, cGAS-STING signaling, immune checkpoint inhibitor, radioiodine therapy, thyroid cancer, tumor immune microenvironment

## Abstract

Radioiodine (RAI) is a cornerstone of treatment for differentiated thyroid cancer (DTC), yet the immunological consequences of RAI-induced DNA damage remain poorly understood. Here, we examine cGAS–STING signaling as a potential mechanistic link between RAI-induced genotoxic stress and antitumor immunity. RAI delivers prolonged, thyroid-selective radiation to iodine-avid tumors, providing a plausible source of extranuclear DNA capable of engaging innate DNA sensing. We propose that transient RAI-associated cGAS–STING activation in DTC may favor IRF3–type I interferon-dependent immune priming, whereas persistent genomic stress in RAI-refractory anaplastic and poorly differentiated thyroid cancers (ATC/PDTC) may shift pathway output toward NF-κB-dominated, myeloid-suppressive programs. This acute-versus-chronic framework suggests that cGAS–STING dynamics could help define a therapeutic window for sequencing RAI with immune checkpoint blockade, while controlled STING activation may provide an alternative strategy for iodine-refractory disease. Direct evidence for RAI-induced cGAS–STING activation in thyroid tumors remains lacking, highlighting the need to define pathway kinetics across thyroid cancer subtypes and determine whether thyroid-specific endocrine signaling, including TSH–TSHR signaling, further modulates innate DNA sensing.

## 1 Introduction

Thyroid cancer is the most common endocrine malignancy worldwide, with an estimated 821,000 new cases and over 47,000 deaths recorded in 2022, and its incidence continues to rise across most regions (1). Despite this growing burden, the disease presents a striking clinical paradox: differentiated thyroid cancer (DTC), which accounts for the majority of cases, generally carries an excellent prognosis, whereas anaplastic thyroid cancer (ATC) and poorly differentiated thyroid cancer (PDTC) remain among the most lethal solid tumors, with disease-specific mortality approaching 100% in unresectable ATC (2). This divergence is closely tied to radioiodine (RAI) responsiveness. DTC retains sodium/iodide symporter (NIS)-mediated iodine uptake and is amenable to RAI-based internal radiotherapy, while dedifferentiation driven by BRAF, RAS, TERT promoter, and TP53 alterations progressively silences NIS expression, rendering PDTC and ATC RAI-refractory (3, 4).

Immune checkpoint inhibitors (ICIs) have recently entered the treatment landscape for aggressive thyroid cancer, with case series and small trials reporting meaningful but inconsistent responses in ATC, particularly when combined with BRAF/MEK inhibitors or tyrosine kinase inhibitors (5). This inconsistency suggests that the immunogenicity of thyroid tumors is shaped by factors beyond mutational burden or PD-L1 expression alone, and that a mechanistic gap remains in understanding what determines whether a given thyroid tumor is immunologically “hot” or “cold” (6, 7).

One underexplored contributor to this gap is the cyclic GMP-AMP synthase-stimulator of interferon genes (cGAS-STING) pathway, a cytosolic DNA-sensing axis that couples genomic instability to innate immune activation and has been increasingly recognized as a determinant of antitumor immunity across multiple cancer types (8). Notably, thyroid cancer possesses a treatment-intrinsic source of DNA damage that is essentially unique among solid tumors: RAI itself, which delivers targeted beta radiation to iodine-avid cells and generates double-strand breaks as part of its therapeutic mechanism (9, 10). Whether this treatment-induced genomic damage engages cGAS-STING signaling, and whether such engagement helps explain the immunogenicity gradient between RAI-avid DTC and RAI-refractory ATC/PDTC, has not been systematically examined.

This mini review therefore focuses on a deliberately narrow question: how does RAI-induced DNA damage intersect with cGAS-STING signaling to shape the thyroid tumor immune microenvironment, and what does this intersection imply for sequencing RAI with emerging immunotherapies. Rather than reprising the tumor microenvironment landscape already covered by recent single-cell and spatial transcriptomic reviews, we build a mechanism-driven, hypothesis-generating framework specific to thyroid cancer’s unique reliance on iodine metabolism and internal radiotherapy.

To inform this narrative mini review, we conducted a focused literature search of PubMed and Web of Science for English-language studies published through August 2026. Search terms included combinations of “thyroid cancer,” “radioiodine” or “radioactive iodine,” “cGAS,” “STING,” “DNA damage,” “cytosolic DNA,” “tumor immunity,” and “immune checkpoint inhibition.” Reference lists of relevant articles were also screened to identify additional studies. Thyroid-specific clinical and preclinical evidence was prioritized where available; when direct thyroid-specific evidence was lacking, mechanistically relevant studies from other tumor types and radiation models were included to support the hypothesis-generating framework.

## 2 Radioiodine-induced DNA damage and cGAS-STING signaling

The cGAS-STING pathway is the principal cytosolic surveillance system for double-stranded DNA (dsDNA). cGAS binds dsDNA that has escaped the nucleus or mitochondria, catalyzing synthesis of the second messenger 2′3′-cGAMP, which activates the endoplasmic reticulum-resident adaptor STING (11). Activated STING recruits TANK-binding kinase 1 (TBK1), which phosphorylates interferon regulatory factor 3 (IRF3), driving its nuclear translocation and transcription of type I interferon (IFN-α/β) and interferon-stimulated genes (12). The established involvement of cGAS–STING signaling in radiation-induced antitumor immunity raises particular interest in thyroid cancer, where radioiodine (RAI) provides a tumor-selective and sustained source of ionizing radiation and DNA damage (13, 14).

RAI (¹³¹I) is selectively concentrated in NIS-expressing thyroid follicular cells, where β-particle emission induces ionizing DNA damage, including DNA strand breaks (15). With a physical half-life of approximately 8.02 days (16), ¹³¹I produces a temporally extended radiation exposure that differs fundamentally from an acute external-beam dose. Importantly, this difference is not merely pharmacokinetic but radiobiological. Unlike external-beam radiotherapy, which delivers a prescribed dose over minutes in discrete fractions, ¹³¹I continuously irradiates iodine-retaining cells at a declining dose rate determined by radioactive decay and biological clearance (17). Consequently, DNA damage is generated over a prolonged interval during which DNA repair, cell-cycle progression, mitochondrial stress, and additional radiation-induced lesions can occur concurrently. Such temporal dispersion may alter both the abundance and persistence of extranuclear DNA available for innate sensing (18). Rather than producing a single burst of cytosolic DNA, RAI could therefore generate a sustained or intermittent DNA-sensing input whose kinetics are determined by the balance between ongoing damage, DNA repair, and clearance of cytosolic DNA. Radiation-induced cGAS–STING activation is highly dose- and fractionation-dependent, reflecting a balance between the generation of immunostimulatory cytosolic DNA and its degradation by nucleases such as TREX1. The protracted, low-dose-rate nature of RAI exposure therefore raises an intriguing parallel with fractionated and low-dose radiotherapy (LDRT), which can remodel the tumor immune microenvironment through cGAS–STING-dependent accumulation of cytosolic nuclear and mitochondrial DNA, dendritic-cell activation, and CD8^+^ T-cell recruitment (19). DNA-damage intensity can determine the immunogenic output of low-dose irradiation. In a triple-negative breast cancer model, radiosensitization combined with PARP inhibition amplified DNA damage under 6-Gy irradiation, activating cGAS–STING signaling and promoting dendritic-cell maturation, macrophage repolarization, and CD4^+^ and CD8^+^ T-cell infiltration (**Figure 1**) (20).

**Figure 1:**
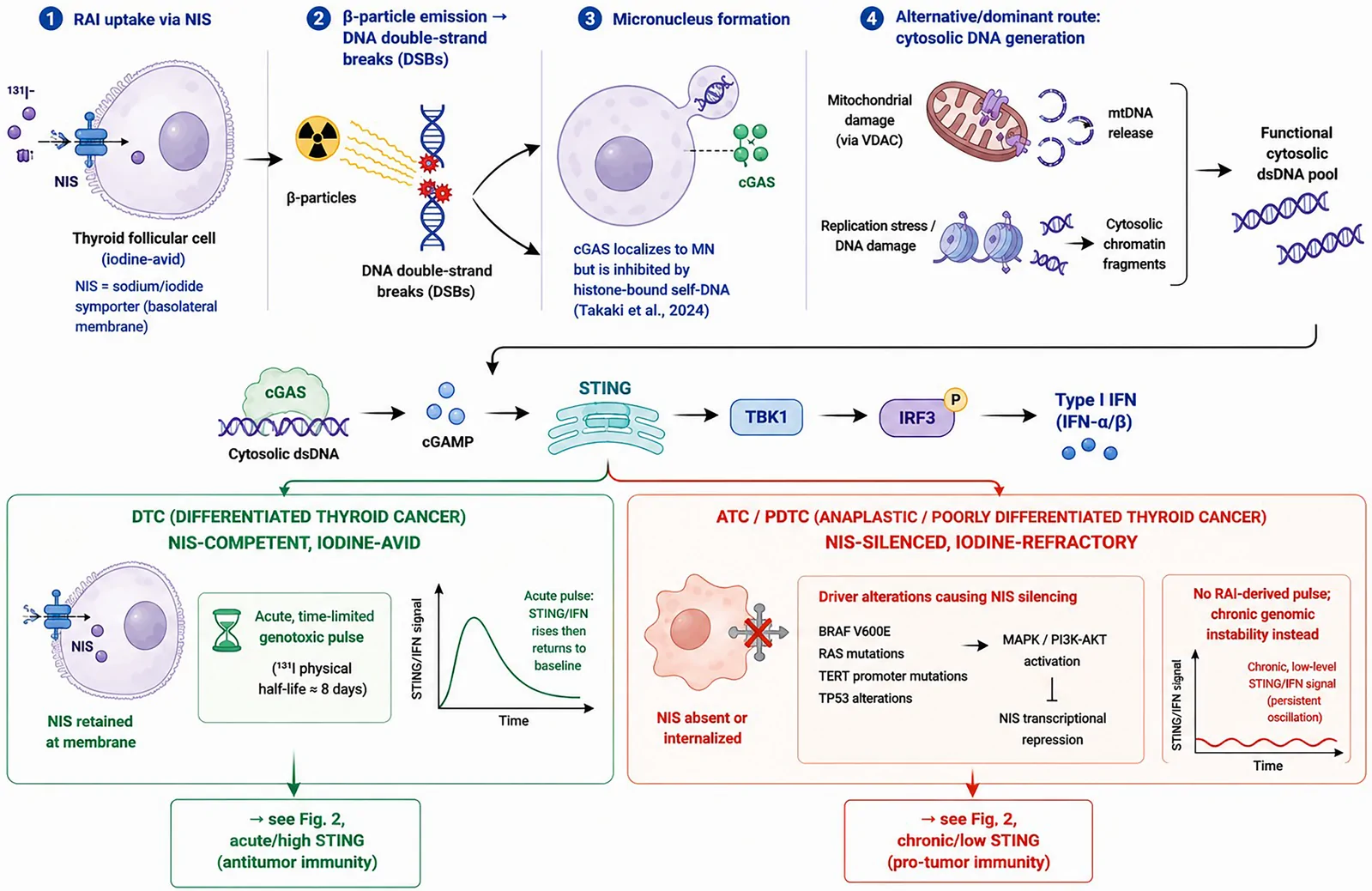
RAI-induced DNA damage and cGAS–STING signaling across thyroid cancer states. Radioiodine (RAI) uptake through the sodium/iodide symporter (NIS) exposes iodine-avid differentiated thyroid cancer (DTC) cells to β-particle-induced DNA damage. Although radiation-induced micronuclei recruit cGAS, chromatinized micronuclear DNA may restrict productive cGAS activation; alternative DNA sources, including mitochondrial DNA (mtDNA) and cytosolic chromatin, may contribute to cGAS–STING–TBK1–IRF3 signaling and type I interferon production. Loss of NIS expression or localization in poorly differentiated and anaplastic thyroid cancers (PDTC/ATC) limits RAI uptake, whereas increased genomic instability may provide an alternative source of endogenous DNA stress. The schematic contrasts a putative RAI-induced DNA-sensing state in iodine-avid DTC with chronic genomic stress in RAI-refractory disease. RAI, radioiodine; NIS, sodium/iodide symporter; DSBs, DNA double-strand breaks; MN, micronucleus; mtDNA, mitochondrial DNA; VDAC, voltage-dependent anion channel; cGAS, cyclic GMP-AMP synthase; cGAMP, cyclic GMP-AMP; STING, stimulator of interferon genes; TBK1, TANK-binding kinase 1; IRF3, interferon regulatory factor 3; IFN, interferon; DTC, differentiated thyroid cancer; ATC, anaplastic thyroid cancer; PDTC, poorly differentiated thyroid cancer.

Radiation-induced micronucleus formation is not necessarily sufficient to initiate productive cGAS–STING signaling. Although cGAS readily accumulates on micronuclei generated by ionizing radiation, replication stress, or chromosome-segregation errors, this localization does not necessarily translate into pathway activation. Takaki et al. showed that such micronuclei failed to induce cGAMP production, STING–TBK1–IRF3 phosphorylation, or interferon-stimulated gene expression, largely because micronuclear DNA remains chromatinized and nucleosome-bound DNA restrains cGAS enzymatic activity (21). These observations argue against a simple DSB–micronucleus–cGAS–STING sequence and suggest that additional forms of extranuclear DNA may be required to sustain radiation-induced innate immune signaling. Mitochondrial DNA represents one such candidate: sublethal mitochondrial outer-membrane permeabilization can release mtDNA through BAX–BAK macropores, thereby activating cGAS–STING signaling (22, 23). More broadly, genomic and mitochondrial stress can generate multiple immunostimulatory DNA species, indicating that the nature, chromatin state and subcellular origin of extranuclear DNA rather than its mere presence are likely to determine whether cGAS becomes productively activated (24). Mitochondrial dysfunction can promote cytosolic mtDNA release and activate cGAS–STING signaling (25). In RAI, this distinction raises the possibility that sustained β-irradiation engages cGAS–STING through a temporally evolving mixture of nuclear and mitochondrial DNA damage rather than through micronucleus formation alone. These considerations raise an important distinction across the thyroid cancer spectrum. As noted above, DTC retains functional NIS expression and is therefore exposed to a treatment-defined, temporally sustained genotoxic stimulus; whether this exposure is sufficient to engage cGAS–STING signaling has not been directly established, though the prolonged generation of nuclear and mitochondrial DNA damage provides a plausible substrate. Progression toward RAI-refractory disease, by contrast, involves not only loss of NIS expression but also impaired NIS membrane localization, both closely linked to oncogenic MAPK signaling and additional genomic alterations acquired during tumor evolution (26, 27).

Loss of RAI avidity does not imply loss of the genomic substrates capable of engaging innate DNA sensing. PDTC and ATC acquire additional alterations associated with dedifferentiation and genomic instability, including frequent TP53 and TERT promoter mutations, and exhibit substantially greater genomic complexity than conventional DTC (28). These features raise the possibility of two biologically distinct contexts for cGAS–STING signaling: a treatment-induced response to sustained genotoxic stress in RAI-avid tumors, and a potentially chronic response to endogenous genomic stress in dedifferentiated, RAI-refractory disease. How these distinct modes of DNA sensing influence immune activation versus immune adaptation remains unresolved.

## 3 Dual effects of cGAS–STING signaling on the thyroid tumor immune microenvironment

Direct thyroid-specific evidence linking cGAS–STING signaling to the proposed acute-versus-chronic framework remains limited. Much of the mechanistic evidence discussed below derives from non-thyroid tumor systems and radiation models. These studies are therefore used to inform a testable hypothesis for thyroid cancer rather than to establish a thyroid-specific mechanism. In this framework, transient, treatment-associated cGAS–STING activation in RAI-avid DTC is contrasted with potentially chronic signaling associated with endogenous genomic stress in RAI-refractory ATC/PDTC.

The immunological consequences of cGAS–STING activation are highly context dependent, ranging from type I interferon-driven antitumor immunity to chronic inflammatory programs that promote immune suppression and tumor progression (**Figure 2**).

**Figure 2:**
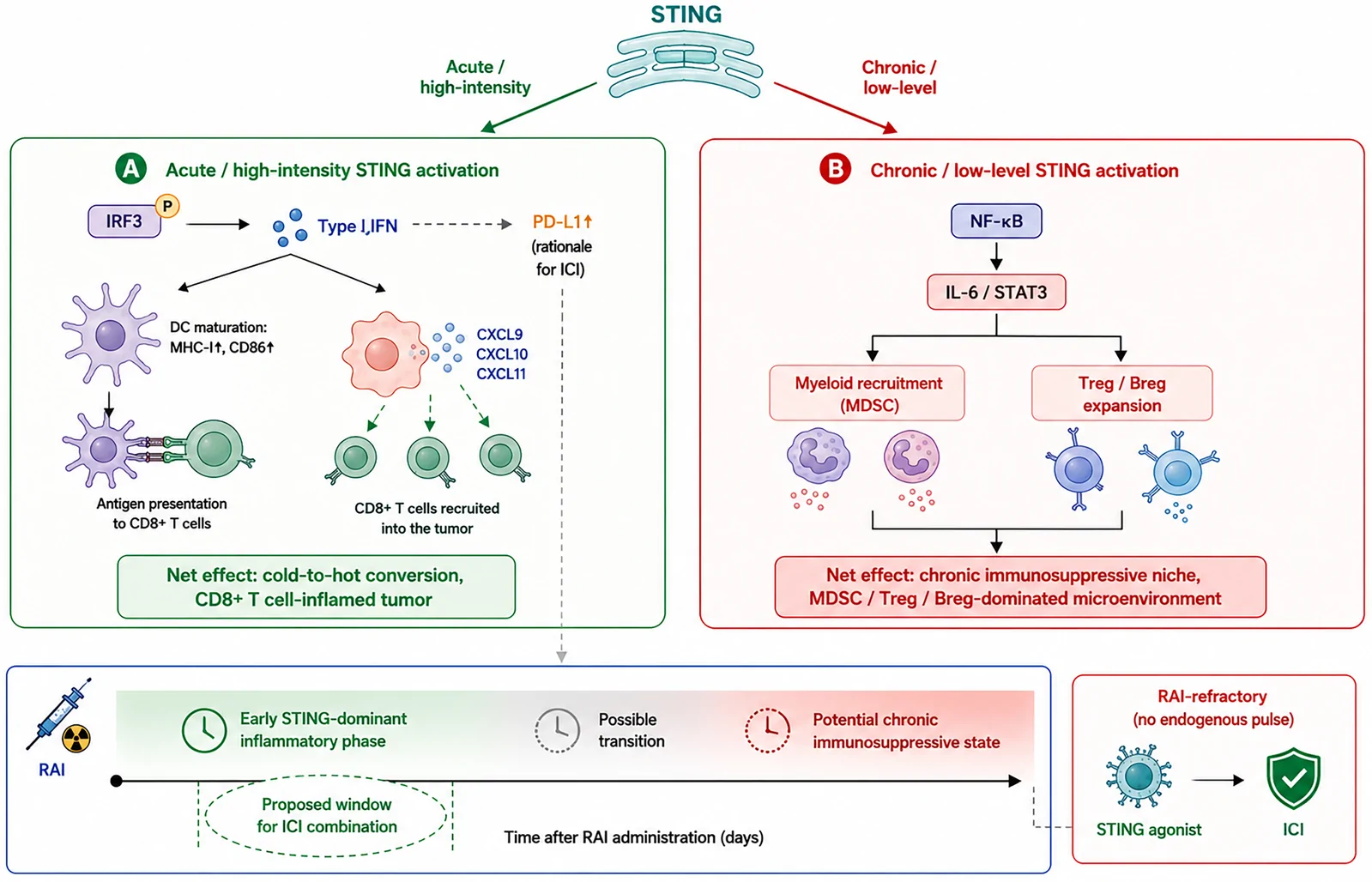
Context-dependent cGAS–STING signaling and a proposed therapeutic window in thyroid cancer. Transient cGAS–STING activation **(A)** favors IRF3–type I interferon signaling, dendritic-cell activation, and CD8⁺ T-cell recruitment, whereas persistent signaling associated with genomic stress **(B)** shifts pathway output toward NF-κB-dominated inflammation and immunosuppressive myeloid programs. The lower panel illustrates a proposed therapeutic framework in which RAI-induced genotoxic stress in iodine-avid differentiated thyroid cancer (DTC) is aligned with immune-checkpoint inhibition, whereas pharmacological STING activation is depicted as a strategy to induce immune priming in RAI-refractory poorly differentiated and anaplastic thyroid cancers (PDTC/ATC). IRF3, interferon regulatory factor 3; DC, dendritic cell; CXCL, C-X-C motif chemokine ligand; PD-L1, programmed death-ligand 1; ICI, immune checkpoint inhibitor; NF-κB, nuclear factor kappa B; MDSC, myeloid-derived suppressor cell; CCL, C-C motif chemokine ligand; CCR2, C-C chemokine receptor type 2; PMN-MDSC, polymorphonuclear MDSC; mtDNA, mitochondrial DNA; Treg, regulatory T cell; Breg, regulatory B cell; RAI, radioiodine.

### 3.1 The antitumor arm

Acute cGAS–STING activation couples tumor-intrinsic DNA sensing to adaptive antitumor immunity through type I interferon (IFN-I) signaling. Experimental enforcement of STING activity in tumor cells induces local activation of antigen-presenting cells, recruitment of effector lymphocytes, and conversion of otherwise poorly immunogenic tumors into an immune-infiltrated state that is responsive to checkpoint blockade (29). This response converges on the dendritic cell–T cell axis: cross-presenting conventional type 1 dendritic cells (cDC1s) are required to generate and sustain broad tumor-specific CD8^+^ T-cell responses, and therapeutic expansion and activation of cDC1s enhances effector CD8^+^ T-cell immunity and tumor control (30). Consistent with this model, pharmacologically induced DNA-repair deficiency in triple-negative breast cancer promoted cytosolic DNA accumulation and cGAS–STING activation, leading to type I IFN–JAK–STAT signaling, increased proinflammatory chemokine production and antigen-presentation programs, and enhanced responsiveness to immune-checkpoint blockade (31).

Cytosolic mtDNA released following VDAC2 loss activates tumor-intrinsic cGAS–STING and type I interferon signaling, whereas disruption of STING pathway components attenuates the resulting antitumor and immunotherapeutic effects (32). Together, these studies position cGAS–STING as a mechanistic bridge between tumor-derived DNA stress, innate inflammatory signaling, and adaptive immune recruitment, providing a rationale for exploiting transient pathway activation to sensitize poorly inflamed tumors to immune-checkpoint blockade.

### 3.2 The pro-tumor arm

In contrast to transient pathway activation, persistent cGAS–STING signaling can be co-opted to sustain tumor-promoting inflammation rather than productive antitumor immunity. A central feature of this transition is a shift in signaling output from an IRF3–type I interferon-dominated program toward NF-κB-dependent inflammatory circuits that favor immune suppression and tumor adaptation. In chromosomally unstable tumors, chronic STING activity has been linked to non-canonical NF-κB signaling and metastatic progression, illustrating how persistent sensing of self-DNA can fundamentally alter the biological consequences of the pathway (33). However, this chromosomal instability–STING–NF-κB axis has not been directly demonstrated in thyroid cancer, and its relevance to ATC/PDTC therefore remains inferential. Metabolic context provides an additional layer of regulation. Lactate was recently shown to bind directly to the cGAMP-binding domain of STING, suppressing STING–IRF3 signaling and cytotoxic immune-cell infiltration; relieving this metabolic constraint restored pathway activity and enhanced responsiveness to PD-1 blockade (34).

### 3.3 Myeloid cells appear to be major effectors of this maladaptive state

Tumor-intrinsic STING signaling can establish chemokine programs that recruit immunosuppressive myeloid populations, including CCR2^+^ monocytic and CXCR2-responsive polymorphonuclear myeloid-derived suppressor cells (MDSCs), thereby converting DNA sensing into a mechanism of immune exclusion rather than immune priming (12). Importantly, this circuitry is not confined to tumor cells. Mitochondrial DNA released from senescent cancer cells can be transferred to PMN-MDSCs, where cell-intrinsic cGAS–STING–NF-κB activation reinforces suppressive programs characterized by ARG1, IL-6, NOS2 and PD-L1 expression (35). Conversely, tumor-intrinsic cGAS–STING signaling can itself reinforce adaptive immune escape. In glioblastoma, OMA1-driven mitochondrial stress increased mtDNA release and activated cGAS–STING, promoting PD-L1 transcription and reducing T-cell cytotoxicity (36). Collectively, these findings depict a feed-forward circuit in which genomically stressed tumor cells and suppressive myeloid populations reinforce one another, converting a surveillance pathway into a driver of immune adaptation.

### 3.4 Context shapes the outcome of cGAS–STING signaling

The divergent consequences of cGAS–STING activation are best understood not as an intrinsic duality of the pathway, but as context-dependent outputs shaped by the magnitude, duration and cellular compartment of DNA sensing. Transient activation preferentially engages IRF3–type I interferon programs that support dendritic-cell priming and adaptive immunity, whereas persistent signaling in the setting of chromosomal instability can undergo functional rewiring toward inflammatory and immunosuppressive states, including NF-κB-dominated and myeloid-associated programs (33). This framework maps directly onto the thyroid cancer spectrum outlined above: cGAS–STING signaling, if engaged, may arise from fundamentally different sources of DNA stress depending on RAI avidity: therapy-induced damage in DTC versus persistent endogenous genomic stress in dedifferentiated, RAI-refractory disease (**Figure 2**) (37).

The immune architecture of aggressive thyroid cancer is compatible with, but does not yet establish, this model. ATC is characterized by prominent infiltration of tumor-associated macrophages, neutrophils and other suppressive myeloid populations, alongside marked intertumoral heterogeneity in lymphoid infiltration. Whether this myeloid-rich state reflects chronic cGAS–STING activity driven by genomic instability, or instead arises through STING-independent consequences of dedifferentiation and oncogenic signaling, remains unknown. Thus, rather than assigning DTC and ATC/PDTC to fixed “STING-active” states, an acute-versus-chronic DNA-sensing framework offers a testable model linking RAI avidity, genomic instability and immune contexture across thyroid cancer progression.

### 3.5 Local immune activation versus systemic immune suppression

RAI also exerts systemic hematologic effects that are distinct from tumor-intrinsic cGAS–STING signaling. Because lymphocytes are among the most radiosensitive hematopoietic cells, circulating ¹³¹I exposes peripheral blood and hematopoietic compartments to protracted low-dose-rate irradiation during systemic clearance (38). In patients with DTC, peripheral lymphocyte populations decline after RAI, with multiple subsets reaching their lowest levels within 30 days and several remaining below pretreatment levels at 90 days, accompanied by persistent alterations in circulating immune- and inflammation-related proteins (39). This systemic lymphocyte suppression may counterbalance tumor-localized immune priming: while RAI-induced DNA damage in iodine-avid tumors may promote innate sensing and T-cell priming, concomitant lymphocyte depletion could limit the adaptive immune response required to sustain antitumor immunity.

This local–systemic tension has important implications for therapeutic sequencing. The net immunological effect of RAI may depend not only on the magnitude and duration of tumor-intrinsic cGAS–STING activation, but also on the depth and recovery kinetics of systemic lymphocyte depletion. An immunologically favorable window for immune-checkpoint blockade may therefore emerge from the temporal balance between local innate immune activation and systemic immune competence. Longitudinal assessment of peripheral lymphocyte counts and subset composition alongside cGAS–STING pathway biomarkers could help define this window.

## 4 Translational hypothesis and open questions

### 4.1 RAI as a potential immunological treatment window

The emerging intersection between radiation-induced innate immunity and thyroid cancer raises the possibility that RAI may influence therapeutic response through mechanisms extending beyond direct tumor-cell killing. Immune-checkpoint blockade has demonstrated activity in subsets of advanced thyroid cancers, including RAI-refractory disease, but responses remain heterogeneous and are incompletely predicted by conventional biomarkers such as PD-L1 expression (40). Importantly, a phase 2 trial in ATC provided prospective evidence that checkpoint inhibition can be effectively integrated with genotype-matched targeted therapy. Among 42 evaluable patients receiving atezolizumab combined with BRAF/MEK-, MEK-, or VEGF-directed therapy according to tumor genotype, median overall survival reached 19 months, compared with a historical benchmark of approximately 5 months; the BRAF^V600E^ cohort treated with vemurafenib, cobimetinib, and atezolizumab achieved a median overall survival of 43 months and a median progression-free survival of 13.9 months (41). These findings demonstrate that even the highly aggressive immune microenvironment of ATC can be therapeutically modulated, although they do not establish whether cGAS–STING signaling contributes to this response. Together, these clinical observations establish the therapeutic responsiveness of the thyroid tumor immune microenvironment, while leaving unresolved whether RAI-induced genotoxic stress creates a transient immune state that could be exploited by checkpoint blockade. To date, no clinical trial in thyroid cancer has specifically evaluated the sequencing of RAI and immune-checkpoint blockade using cGAS–STING pathway biomarkers, and the proposed therapeutic window therefore remains hypothesis-generating.

### 4.2 Therapeutic manipulation of cGAS–STING in RAI-refractory disease

Loss of iodine avidity presents a fundamentally different therapeutic context. PDTC and ATC lack the NIS-dependent genotoxic stimulus provided by RAI, yet frequently exhibit genomic instability and a complex, myeloid-rich immune microenvironment. Pharmacological activation of STING therefore represents a conceptually attractive strategy for restoring transient innate immune priming in tumors that cannot generate an RAI-associated DNA-damage response. Across non-thyroid tumor models, STING agonism enhances antigen presentation, effector T-cell recruitment and responsiveness to immune-checkpoint blockade; however, clinical translation has been constrained by limited intratumoral delivery, systemic inflammatory toxicity and the context-dependent consequences of sustained pathway activation (42, 43). Importantly, thyroid-specific preclinical evidence has now begun to emerge. Tumor-targeted co-delivery of cGAMP and a TGF-β inhibitor enhanced STING signaling, promoted dendritic-cell maturation and CD8^+^ T-cell infiltration, reduced regulatory T-cell abundance, and suppressed thyroid tumor growth (44).

These limitations are particularly relevant to aggressive thyroid cancers. Rather than assuming that greater STING activity is uniformly beneficial, therapeutic strategies may need to favor transient, tumor-localized pathway activation while avoiding prolonged signaling that could reinforce myeloid suppression. Whether STING agonism can achieve this balance in PDTC or ATC remains unknown and warrants evaluation in thyroid-specific preclinical models before clinical translation.

### 4.3 Biomarkers and unresolved biology

Translating this framework will require biomarkers that capture cGAS–STING dynamics rather than pathway abundance alone. Static expression of cGAS–STING pathway components may not reliably reflect productive immune signaling or therapeutic responsiveness (45). Longitudinal interferon-stimulated gene signatures could provide a systemic measure of pathway output before and after RAI, whereas circulating cGAMP and cell-free DNA represent potential proximal indicators of DNA sensing (46, 47). Where post-treatment tissue is accessible, single-cell profiling or multiplex imaging could resolve TBK1–IRF3 activation together with dendritic-cell and T-cell recruitment within the tumor, thereby distinguishing local pathway engagement from the systemic lymphocyte dynamics described above. Integrating these temporal and spatial measurements could establish whether ¹³¹I induces a reproducible innate immune response, define its relationship to adaptive immune recruitment, and determine whether cGAS–STING activity can serve as a pharmacodynamic biomarker for sequencing RAI with immune-checkpoint blockade.

Fundamental biological uncertainties nevertheless remain. Direct evidence that therapeutic RAI activates cGAS–STING in human thyroid tumors is still lacking. The identity and cellular origin of the immunostimulatory DNA generated after RAI, the relative contributions of tumor and stromal compartments, and the extent to which pathway dynamics differ between iodine-avid DTC and dedifferentiated PDTC/ATC remain unresolved. Addressing these questions will determine whether the proposed RAI–cGAS–STING axis represents a therapeutically actionable feature of thyroid cancer or reflects the broader innate response to radiation-induced DNA damage.

### 4.4 Endocrine modulation of cGAS–STING signaling

A largely unexplored question is whether thyroid-specific endocrine signaling modifies cGAS–STING activity independently of its effects on DNA damage. TSH–TSHR signaling is a central regulator of thyroid differentiation and NIS expression and is deliberately manipulated during the clinical use of RAI, through endogenous TSH elevation or recombinant human TSH stimulation before treatment and subsequent TSH suppression during long-term management (48). Whether TSHR signaling, or the broader iodine-handling program of the thyrocyte, directly alters the expression, activation threshold, or downstream output of cGAS–STING signaling remains unknown. Such regulation could influence not only the magnitude of RAI uptake but also the cellular response to the resulting genotoxic stress, potentially coupling endocrine state to innate immune sensing. Defining this interface may therefore reveal whether RAI-induced immune signaling is determined solely by radiation dose and DNA damage or is additionally conditioned by the differentiated and hormonal state of the thyroid cell.

## 5 Conclusion and future perspectives

Thyroid cancer occupies a distinctive position in oncology because tumor differentiation directly determines exposure to a treatment-intrinsic source of ionizing radiation. This coupling between iodine avidity and radiation-induced genotoxic stress provides a unique setting in which to examine how the magnitude, duration, and cellular origin of DNA damage shape innate immune sensing. We propose that RAI-avid and RAI-refractory disease represent mechanistically distinct states of cGAS–STING engagement: acute and treatment-induced in DTC versus chronic and endogenous in dedifferentiated PDTC/ATC. Establishing whether these states exist in patients could provide a mechanistic basis for integrating RAI, immune-checkpoint blockade, and STING-directed therapies.

Beyond thyroid cancer, this framework may extend to other forms of targeted radionuclide therapy. Radioligand and peptide receptor radionuclide therapies similarly expose target-expressing tumors to sustained internal irradiation, yet the consequences for innate DNA sensing and antitumor immunity remain incompletely defined (49). The unusually tight coupling between differentiation, radionuclide uptake, and therapeutic response in thyroid cancer may therefore provide a tractable model for understanding how internal radiotherapy intersects with innate immune sensing more broadly.

Realizing this translational potential will require resolving local and systemic immune dynamics over time. RAI-induced genotoxic stress within the tumor occurs alongside systemic lymphocyte depletion, making its net immunological effect a balance between local innate immune priming and systemic immune competence. Defining the kinetics of these processes and their relationship to adaptive immune recruitment will be essential for determining whether RAI creates a therapeutically exploitable immune window and, ultimately, whether cGAS–STING can be translated from a mechanistic hypothesis into a biomarker-guided strategy for thyroid cancer.
